# Friendship in Later Life: How Friends Are Significant Resources in Older Persons’ Communication about Chronic Pain

**DOI:** 10.3390/ijerph19095551

**Published:** 2022-05-03

**Authors:** Imane Semlali, Gilles Merminod, Orest Weber, Ana Terrier, Isabelle Decosterd, Eve Rubli Truchard, Pascal Singy

**Affiliations:** 1 Liaison Psychiatry Service, Department of Psychiatry, Lausanne University Hospital, Av. De Beaumont 23, 1011 Lausanne, Switzerland; gilles.merminod@unil.ch (G.M.); orest.weber@chuv.ch (O.W.); anamaria.terrier@unil.ch (A.T.); pascal.singy@chuv.ch (P.S.); 2 Pain Center, Service of Anesthesiology, Lausanne University Hospital, 1011 Lausanne, Switzerland; isabelle.decosterd@chuv.ch; 3 Geriatrics and Geriatric Rehabilitation Service, Department of Medicine, Lausanne University Hospital, 1011 Lausanne, Switzerland; eve.rubli@chuv.ch

**Keywords:** older persons, chronic pain, communication, friendship, social network, emotional support

## Abstract

Background: This article focuses on how older persons perceive their friends’ role in their daily experience of chronic pain. It reports part of the results of a study in which we interviewed 49 participants, aged 75 and older, about the way they communicate about chronic pain within their social network. Methodology: Using discourse and content analysis, we first examine older persons’ definition of friendship, and then identify the various dimensions of friendship that are engaged in the communication about chronic pain. Results: Participants define close friends as people with whom they share intimacy and social proximity (same gender, age and experience of pain). These dimensions allow older persons to talk freely about their pain without the fear of being judged or rejected, particularly when it is related to a dynamic of reciprocity. Conclusions: This article shows that the contribution of friends to the everyday life of older persons with chronic pain is mainly that of providing emotional support.

## 1. Introduction

A social network is often described as a significant determinant of physical and psychological health [1,2,3]. In later life, having a diverse network (e.g., family, friends, caregivers) is a predictor of better health outcomes, lower morbidity and mortality [4,5,6] and well-being [7]. The existence of social support, such as material assistance or emotional help, is beneficial for the health of older persons, either directly through physiological responses to stress or emotional regulation or indirectly through better access to services and resources [4,8].

In later life, events such as losing relatives and friends, chronic illness or relocation to a retirement home can disrupt the structure of the social network [3,9]. Friends, while playing a key role in maintaining social ties, are the most threatened by this remodeling [10]. In addition, the likelihood of making new friends in later life is lower than in adulthood [3]. Unlike the family bond, the friendship bond is based on the interest of individuals and is more likely to be broken by a lack of reciprocity [10].

Like most Western countries, Switzerland has been experiencing a significant ageing of its population for several decades, accompanied by an ever-increasing longevity [11]. A consequence of this demographic evolution is that the medical world is confronted with new challenges, particularly concerning chronic health problems related to advanced age [12]. Switzerland is culturally marked by the principle of individualism. Older persons are therefore encouraged to be autonomous, in contrast to more familial cultures, where the older persons can count on the material and financial support of their relatives [13]. To encourage this principle of autonomy, older persons’ specific structures in Switzerland are designed to meet their health and social needs and to promote their autonomy (home care, meeting facilities, associations, retirement homes, etc.) and maintain social ties. Most of them are financed by the social security system, so that they do not depend on their families. 

Although many authors have studied family structure and its impact on older persons’ health management [14,15,16], the role played by friends in their everyday life still remains underexplored. In this context, we will try to show how friends play a significant role in the older persons’ well-being and management of pain. To answer this question, we focus on the particular case of chronic pain, which is an important factor of vulnerability among older persons [17] and impacts on the whole social environment of the people who suffer from it [18,19]. This article will focus on the way older persons define friendship. Based on an inductive approach, we chose not to give a prior definition of friendship but to base our study on the participants’ own definition, or definitions, and then identify the parameters at stake in the communication of chronic pain in such a relational setting. 

## 2. Methodology

### 2.1. Study Design

This article is part of a larger study on the communication about chronic pain within older persons’ social networks. The theoretical foundation of this research is based on a pragmatic and sociolinguistic perspective, which rests on the fact that communication is more than an exchange of information. Communication also allows individuals to act on the social world, to modify it and thus achieve practical or relational objectives [20]. This research is also based on personal communication networks theory, where the network is studied from the perspective of a focal actor [21].

A multidisciplinary research team (linguistics, psychology, medicine) interviewed older persons suffering from chronic pain. The goal was to study how older persons perceive the communication about chronic pain within their personal network and to bring out their specific needs. 

To analyse the interviews, we combined discourse and content analysis with a qualitative social network analysis [22]. A steering committee (with medical and nursing staff, experienced researchers and decision-makers working on later-life issues) accompanied the research team throughout the project.

### 2.2. Data Collection

The recruitment of participants was carried out with the help of institutions (day centres, retirement homes, pain clinics, associations, home care) and individuals working with older persons (members of the research team and members of the steering committee). We also used the snowball technique (indication of new respondents by the participants). Our sample consisted of 49 individuals from the French-speaking part of Switzerland, all suffering from chronic pain. We defined chronic pain as pain lasting more than three months [23]. All the participants were aged 75 and over. None of them had major cognitive or auditory impairments. We constructed our sample to take into account the socio-demographic variations within the older population (Table 1). Older persons are diverse, and the way they communicate about health is impacted by their life trajectories and social affiliations [24]. 

Four members of the research team conducted semi-structured interviews [25,26]. The interview guide was refined during this phase [27]. The face-to-face interviews brought together one researcher with one older person at the interviewee’s home or in our office if the interviewee so wished. In order to gain as much information as possible from the interviews, we met each participant twice. The first time (45–60 min), the aim was to get to know the older persons by collecting socio-biographical data (brief life history) and to get them to describe the pain from which they suffer (type, duration, intensity, management, functional limitations, emotional and relational impact). The participants were also asked to report on their social network and to describe the type of ties it contains. In order to collect this information, we used the concentric circle method [28]. With this method, the participants can map their personal network and arrange its members according to their degree of importance. The second time we met the participants (45–60 min), generally a few days later, we discussed in more detail the way the older persons communicate about chronic pain with each member of their network (frequency of exchanges, content and goals, difficulties and preferences, motivations and consequences, specific strategies for initiating or avoiding communication about chronic pain). We also explored the older persons’ specific communicative and informational needs and expectations.

### 2.3. Data Analysis

More than 95 interviews were audio-recorded and transcribed. The research team coded the interviews using content analysis [29,30] completed by discourse analysis [31]. Such a method of analysis enabled us to identify the semantic categories the participants consider relevant to describe how and why they communicate about chronic pain within their social network. The coding followed an inductive logic, relying on a process of intercoder agreement. Coding was carried out with Nvivo, which is a software package that allows for the systematisation of qualitative analysis [32].

## 3. Results

Our data show the existence of three subtypes of network within the social network in which it is possible to talk about health issues and illness-related difficulties: health professionals, relatives and friends. The first two are strongly determined by the function of the relationship (health professionals: transactional function) or its nature (relatives: relationship of kinship or alliance). What determines the friends’ network is subject to greater variation depending on the respondents. However, the data analysis indicates that there is a consensus among the participants on the definition of what constitutes friendship, despite a heterogeneity in the terms employed (e.g., friends, buddies, pals) and the meaning associated with them (from strong ties to weak ties). 

### 3.1. Intimacy

Participants generally described friendship using the parameter of intimacy, contrasting close friends with acquaintances. These two categories are the two poles of a continuum that varies according to the lexical categories employed (e.g., friends, close friends, distant friends, intimates, acquaintances, mates) and the semantic investment made by the participants in the study.

“Among our close friends, they know that I have these problems. We talk about it from time to time but I don’t want to burden people with my problems”.

(W75 = Woman, 75 years old)

Close friends are differentiated by the voluntary sharing of their privacy and by a form of reciprocity. When it comes to chronic pain, which is considered a private matter, our data showed that participants talk far more easily about pain with some of their friends whom they consider to be intimates:

“Anyway, we’ve been friends for over 50 years. We tell each other everything, and the good thing is that it stays with us, it doesn’t go anywhere else. We confide in each other but it doesn’t go anywhere else”.

(W87)

Duration and mutual trust seem to be the two main dimensions that are associated with intimacy and facilitate conversations about pain. Such a relationship, therefore, allows for a disclosure that is not jeopardised by the risk of circulation of the remarks outside a situation of communication.

Relationships with acquaintances, on the other hand, are characterised by the silencing of private problems. Our respondents tend to avoid talking about their pain with people whom they feel they do not share sufficient trust and intimacy.

“We have friends like everyone else. But they are the type of friends we see from time to time. We share a meal or something like that. But they’re not close friends. I’m not going to call them to complain about something”.

(M76 = Man, 76 years old)

For some of the interviewees, it seems inconceivable or, at the very least, inappropriate for an acquaintance to talk about the issue of chronic pain:

“If, for example, someone I know, who is not very close to me, [asks me about my pain], I ask myself: ‘But why is she asking me these questions? Why is she interested in me?’”

(W82)

By staging her perplexity, the participant shows the extent to which such a thematic would disturb social rules that generally remain tacit.

The participants in the study describe an acquaintance as a person belonging to a so-called secondary network, which may include former colleagues with whom they have maintained sporadic contact, as well as people who belong to the same associations (e.g., sports, culture and politics) or religious groups. We can also add co-residents of the institution, who have the particularity of also being in a neighbourhood relationship. In our data, the relationship between residents appears to be no more than simple co-presence. There does not seem to be any real cooperation between the participants that would verbally maintain a joint activity. Despite frequent encounters, the quality of the relationship is not perceived as being strong enough to address the issue of chronic pain. This is well illustrated by one quote from a woman talking about her relationship with the other residents:

“With these people here, it’s difficult, because I can’t tell (…) I can’t, it’s too difficult, it’s too personal”.

(W86)

For the participants who are still living in their homes, neighbours are often mentioned as acquaintances, who may sometimes be essential to the smooth running of their daily lives. Although spatial proximity could facilitate communication, neighbours rarely attain a degree of intimacy that allows for the discussion of private matters.

“My neighbours, in the house, well, when I meet them like this: “Hello, are you all right?” “I’m fine” “Yes, yes, I’m fine”. I’m not going to say: “Listen, this morning, damn, my shoulder hurts””.

(W82)

The relationship can be of material or logistical assistance (e.g., doing the shopping, bringing in the mail). This mutual aid, even when it relates to difficulties linked to the health problems of the interviewees, does not, however, lead to any communication about chronic pain. Neighbours are, therefore, not considered as interlocutors when it comes to pain, even when they show an interest in the one who suffers. In the same way, their help with everyday tasks does not change this state of affairs. However, social networks are multiplex, and some neighbours have become intimates with whom it is possible to discuss chronic pain. They often share the same social profile (gender, age, experience of pain). Friendships are more likely to evolve over time compared with other interpersonal ties, even in later life. Neighbours can become friends and, in some situations, even close friends.

“… and then there are my little neighbours who are also very important (…) and we have a lot of contact, I see them once or twice a week (…) and he comes to vacuum once a month and I have very good connection with them, it’s just that I didn’t think about it because they have become a bit like our adopted children”.

(M87)

### 3.2. Social Proximity

According to our data, intimacy goes hand in hand with social proximity and facilitates communication about pain. In the data, respondents portray intimates with the same socio-biographical profile (similar gender, age, background, social class, etc.).

“With our group of friends, we’ve been together since 1973 and we’re still the same people, growing old together, having babies, children, and grandchildren. Now we live alone. We have all kinds of pain, and then obviously we can talk to each other without any problem, because everyone has something… or almost”.

(W75)

According to the participants, being of the same age plays an important role in this feeling of commonality. This fact is well illustrated by the following extract in which one of the participants specifies to whom, in particular, she talks about her health difficulties:

“Especially with friends my own age, but also almost always with people who are by my side since ’91, who have known me since then, who know that things are not going so well.”

(W75)

The old age of the participants does not seem to be enough to facilitate the communication. In our data, the quality of the relationship is correlated with its duration, as already mentioned.

Sharing the same gender identity can, at least for women, act as a facilitator for communication about chronic pain. Our data show that women are interlocutors for other women when talking about health problems, as illustrated by the following extract:

“Yes, yes, because we tell each other everything, I feel that she is understanding. So it feels good to feel understood”.

(W83)

The data do not show women making this type of comment about male friends.

Another form of social proximity that emerged from our data is the shared experience of pain. The experience of pain is always singular, but sharing the same problems makes inter-understanding and reciprocity of perspective possible:

“Ah, with Suzanne (alias) too, that’s for sure. She’s always had back problems, she’s had several operations, she knows what it’s like. With her, I can talk”.

(W80)

The shared experience may be specific (e.g., back or knee pain), as shown in the following extract:

“(I talk) with Marie-José (alias), because she is going to have a knee operation. I’ve also had a knee operation. So, yes, we are talking about that”.

(W91)

It can also be more general and correspond to a comparable painful experience without the same health problems, as indicated in the following extract:

“There’s Lily (alias), yeah. But she’s had a bit of the same journey as me, lots of problems, lots of pain”.

(W80)

In the same line, some of the interviewees emphasise the difficulty that people without chronic pain have in understanding the experience of suffering.

“Someone who is doing very well, who has everything going well, he doesn’t want to talk about it. For him, it’s not important, you see”.

(W82)

Older persons may perceive such interactions as potentially risky. It could threaten the relational balance between individuals.

“Without me saying anything, they are now more willing to help me carry, they are much more attentive. But I think that if I had got on their nerves I might not have seen them so much”.

(W84)

What emerges here is the issue of not being seen as the eternal complainer. Also evident is the issue of not giving friends the impression that they are just being instrumentalised as the receivers of complaints. 

## 4. Discussion

Despite some variations in what friendship means, the results show a consensus among the participants. It is primarily intimacy that distinguishes close friends from other types of friendship (acquaintances, buddies, etc.) and allows for the expression of pain [1]. Intimacy is strengthened by several factors, such as the long duration of the relationship and mutual trust [33]. Intimacy can only develop through the will of the people involved in the relationship, which is why the link between the two cannot be built artificially nor forced [34]. In addition to intimacy, social proximity, understood as a similarity in age, gender and experience, leads to a form of community of fate [35].

Many studies about friendship underline the complexity involved in forming a proper definition of this phenomenon and explaining the process that gives rise to this kind of social connection [1,35,36]. However, some authors mention concepts that are similar to our results, relating specifically to the definition of friendship, particularly in later life [3,34]. They also bring out the emotional bond as the main factor that distinguishes friendship from other relationships (acquaintances, strangers, etc.).

Intimacy and social proximity refer to an exchange between two friends and imply a dynamic of reciprocity [3,10]. In later life, this reciprocity is more prominent because of the tendency to homophily that appears to be a major feature of friendship [37,38,39]. Indeed, attached to a shared history and experience, the similarity between friends enables a form of balance between interlocutors, both of whom can express their own difficulties and listen to the other [40]. The communication about pain is, therefore, linked to a mechanism of reciprocity that enables the person who is suffering to be more than just a complainer and, as a consequence, indebted (in terms of time and attention) [41]. They can be a hearer and a support. The fact that the interlocutor shares a similar experience prevents, in a way, the asymmetry that could be built up when revealing one’s own weaknesses [41]. In addition to avoiding a possible relational instability and thus saving face [41,42], a similar experience of pain, for many of the interviewees, appears to be a necessary condition for genuine communication about this topic [43]. More than an exchange of information, the communication of one’s pain thus becomes a communion, echoing the inexpressible nature of the pain experienced in the flesh and exceeding the power of words alone [40].

The data analysis shows that a shared experience, or commonality, strengthens friendship through a dynamic of reciprocity. We can take this further and ask whether sharing a similar experience can be a vector for the formation of friendships. While the data collected do not provide evidence of people becoming friends because they share similar health problems, the literature indicates it does occur in certain settings, such as support groups [44,45].

To sum up, whatever the nature of a friend’s presumed inability to be an adequate interlocutor, it will often be linked to a difficult-to-achieve reciprocity, as is frequently the case for people living in institutions who are not necessarily inclined (because of physical, psychological or cognitive problems) to communicate about chronic pain. In such a context, it should be noted that the relationship between individuals is not chosen but somewhat forced, in the sense that individuals find themselves in contact by default [46].

Research on social networks shows a division of labour between the different stakeholders within the social network [47]. Family members are more involved in the daily needs of older persons and health professionals are more involved in the treatment of pain, while friends are important for emotional support [48]. We noticed that friends are critical in providing emotional support, which is an important resource for psychological well-being [49,50]. The literature mentions two types of support: general support and targeted support [44]. The former refers to supportive behaviour under normal circumstances, and targeted support refers to supportive behaviour that is reactive to specific events, such as health problems. It appears that people need to establish a general supportive relationship before they can perform any type of targeted support [44]. Therefore, in order to provide emotional support to a friend with chronic pain, general support must first be established reciprocally within the relationship.

Intimate friendship allows for a dynamic of confidence, which is only possible when the relationship is based on mutual trust [40]. Confidence is a risky activity for the person who confides and the person who plays the role of confidant. On the one hand, the former is in a position of weakness by testifying to his/her suffering. On the other hand, the confidant sees his/her range of action reduced to listening to the other’s story [51]. Nevertheless, the very fact that a reversal of roles is possible—relating to the imperative of reciprocity—keeps the relationship equal. This dynamic of trust allows for mutual emotional support [48].

The importance of such a level of equality and reciprocity for self-disclosure is well illustrated when one considers the reverse examples of asymmetrical relationships (e.g., parent–child or doctor–patient) in which one of the parties is the carer and the other one the cared-for [41]. In this kind of relationship, the inequality in status could weigh on autonomy [37]—the fear that the interlocutor will adopt a paternalistic position and make decisions for the older persons (e.g., placement in a nursing home). On the other hand, injunctions and advice from friends may be seen as particularly unwelcome [52]. To sum up, emotional support is presented as the primary contribution of friends when discussing pain. Emotional support provided by friends makes it easier to manage pain [53]. Put simply, friendship helps older persons to live with pain.

### Implications

Although the family is the focus of many studies on social support in later life [3], it now seems necessary to examine the involvement of other members of the social network (especially friends) [50] because, in some contexts, family no longer has the central role in the care of the older persons [5]. The relationship with friends offers a space in which one can speak freely, which is different from the relationship with family or health professionals. Some studies show that friends have more positive effects on the psychological well-being of older persons (increased self-esteem, reduced feelings of isolation) than family [50,54]. Friendship is a matter of choice. Friends may be more open to conversations of mutual interest that are oriented towards the enhancement of a positive effect [48]. Our data seem to confirm the importance of close friends for the physical and psychological health of older persons. In a context of care, it would be necessary for health professionals to value relationships where the communication of health problems is facilitated, which would be the gateway to better care. So far, the collaboration between relatives and health professionals is preferred, and clinicians tend to see friends as subsidiary to relatives in a care setting. They should try to see friends’ support as being complementary to that of relatives. Furthermore, it would also be relevant to include close friends in care if it corresponds with the older persons’ wishes. 

Nevertheless, older persons generally experience a disruption in the structure of their network due to the loss of close and distant friends, followed by a difficulty in renewing their network [55]. This leads to an increase in social isolation, with a consequent decline in health and well-being [8,46]. A reduction in the frequency of contact can cause isolation and feelings of loneliness [56] however, a perceived lack of interpersonal intimacy should not be underestimated either [46]. In some ways, the reduction of the network, especially the absence of intimates, affects the emotional support of older persons with health problems. It is essential, therefore, to further investigate the relational dynamics of close friends and their involvement in the health and well-being of older persons [3]. This investigation could lead to the development of campaigns that aim to increase the importance of maintaining a network of close friends from an early age. It is also important that caregivers bear in mind what is at stake in the field of friendship in order to be able to approach the older patient in a truly holistic manner (patient-centred medicine, family and friends, etc.). They could then understand what resources are available in terms of emotional support for managing their health problems on a daily basis [49,57].

In spite of the scope of our study in relation to the numbers of participants and the methodology of investigation (mixing the social network theme with content and discourse analysis), our results are not without limitations. Firstly, our research focused on the participants’ personal point of view about their interactions. In order to analyse the whole relational ecology, further research should investigate all actors involved in these interactions. Secondly, the results of our research are limited solely to the practices that participants reported. Long-term fieldwork would be needed to find out how friendship is actually experienced every day by older persons with chronic pain. Thirdly, we excluded participants with major auditory and cognitive issues for practical reasons. This population implies specific issues that deserve further investigations. Finally, we have highlighted the opinions of a limited population from a socio-spatial point of view (French-speaking part of Switzerland). We can, therefore, ask ourselves the question, what is happening in other parts of the world?

## 5. Conclusions

This study allowed us to investigate how older persons perceive the role of friends in their daily life with chronic pain. We found that the experience of pain was easier to communicate with close friends who share a social proximity and whose relationship is based on reciprocity. We were thus able to highlight that friends are an important socio-affective resource for the health of older persons. For the medical field, this study gives access to the private world of patients. This research is an opportunity for health professionals who do not usually have access to such data to look at the relational dynamics at play in the life of those they care for.

## Figures and Tables

**Table 1:** Respondents’ socio-demographic details.

Variables		
**Gender**	Woman Man	34 15
**Age**	75–85 More than 85	28 21
**Primary Place of Socialisation**	Switzerland Other	34 15
**Socio-Economic Level**	Lower social class Upper social class	31 18
**Residence**	Home Nursing home	40 9
**Descendant**	Yes No	39 10
**Relationship Status**	Single or widowed With partner	33 16

## Data Availability

The data presented in this study are available on request from the corresponding author. The data are not publicly available due to privacy reason.
